# Evaluation of preoperative ACE inhibitors for renal protection in cardiac surgery

**DOI:** 10.1097/MD.0000000000037760

**Published:** 2026-03-27

**Authors:** Yasin Kilic, Izatullah Jalalzai, Ebubekir Sonmez, Bilgehan Erkut

**Affiliations:** a Department of Cardiovascular Surgery, Atatürk University Medical Faculty, Erzurum, Turkey.

**Keywords:** ACE inhibitors, cardiac surgery, coronary artery disease, postoperative, renal function

## Abstract

**Background:**

There are many causes of acute kidney injury after cardiac surgery. Activation of the renin-angiotensin system is one of the most important mechanisms. In this article, we investigated the effect of angiotensin-converting enzyme (ACE) inhibitor treatment on renal damage in cardiac surgery.

**Methods:**

Six hundred twelve patients undergoing cardiac surgery were divided into 2 groups. The patients in group I were 319 patients who used ACE inhibitors before the operation. The patients in Group II consisted of 293 patients who did not use ACE inhibitors. In addition to blood renal parameters and the need for dialysis, length of hospital stay, hospital costs, and readmission rates were evaluated between the 2 groups. In addition to the early-term results, the patients were followed for 2 years for renal destruction.

**Results:**

Dialysis was needed in 9 patients in Group I. Hemodialysis was required in 31 of the Group II patients. In addition, the increase in creatinine and blood urea nitrogen levels and the decrease in glomerular filtration rate and creatinine clearance were higher in Group II patients (*P* <.05). The length of stay in the intensive care unit and hospitalization were statistically significantly higher in Group II patients. In addition, hospital costs and re-hospitalizations were statistically significantly lower in the treatment group.

**Conclusion:**

The use of ACE inhibitors before cardiac surgery is effective in preserving renal function in the postoperative period, and these results are important in achieving acceptable and favorable outcomes for patients and the hospital in the postoperative period.

## 1. Introduction

Multiple organ failures are a common cause of morbidity and mortality after cardiac surgery. Cardiopulmonary bypass (CPB) established during cardiac surgery may cause organ damage and dysfunction, and the most important known cause of this is the presence of endogenous vasoactive agents and inflammatory mediators.^[1,2]^ CPB may initiate renal damage by causing an increase in renal resistance, a decrease in renal blood flow, and a decrease in glomerular filtration rate.^[3]^ The rate of renal damage has been shown to be up to 30 % in many studies.^[3,4]^ Angiotensin II, released from the moment CPB is initiated, begins to act as one of the most highly potent vasoactive agents, impairing the perfusion of the viscera and regional oxygen utilization. In addition to its vaso-constrictive effect on macro- and micro-vessels, it increases the release and effect of other endogenous vasoconstrictors such as catecholamine and endothelin. In addition, it inhibits the release of vasodilators from the vascular endothelium, including nitric oxide and prostacyclin.^[1,5]^

Angiotensin-converting enzyme (ACE) inhibitors have been widely prescribed over an extended period for the management of hypertension and heart failure. Chronic heart failure patients who take these medications have a lower risk of organ malfunction, less cardiovascular system damage, and a longer life expectancy. ACE inhibitors reduce the vaso-constrictive effects of angiotensin II and may increase macro- and microcirculatory blood flow in addition to promoting the vasodilator activity of tissue bradykinin and prostaglandins. Relatedly, it may offer protection against CPB-induced ischemia and reperfusion injury.^[1–3]^

ACE inhibitors can increase kidney damage during CPB-related hypo-perfusion and lead to a temporary decline in renal function due to their effect on reducing glomerular perfusion pressure. Despite this negative effect, it has been shown in many series and studies that ACE inhibitor therapy provides renal protection in different clinical settings.^[1–8]^

The purpose of this study was to assess the hypothesis that administering ACE inhibitors following cardiovascular surgery, including CPB, may provide beneficial effects on renal function, postoperative parameters (including mortality, length of hospital stay, readmission rates, and hospital expenditures), and overall clinical outcomes.

## 2. Methods

Between November 2017 and October 2023, 612 patients who underwent cardiac surgery in our clinic were included in the study. Patients were divided into 2 groups. Patients in Group I (*n* = 319) who received ACE inhibitor treatment at least 1 month before surgery and patients in Group II (*n* = 293) who did not receive any ACE inhibitor were compared in terms of acute kidney injury (AKI) and various parameters. The following parameters can be counted among the factors compared between the groups in the postoperative period: In the early postoperative period, drug therapies, biochemical data indicating renal function (blood urea nitrogen, creatinine, creatinine clearance, glomerular filtration rate), dialysis requirement, bleeding and bleeding-related revision rates, extubation time, hospital and intensive care unit (ICU) length of stay, operative mortality, organ dysfunctions and infections, hospital and ICU length of stay, hospital costs, and in the late postoperative period, patient loss, hospital readmission, and dialysis requirement.

Group I patients had been receiving oral treatment with Silazapril (INHIBACE® 1–2.5–5 mg), Ramipril (DELIX® 2.5–5–10 mg), Enalapril (ENAPRIL® 5–10–20 mg), and Lisinopril (RILACE® 5–10-20 mg) for at least 4 weeks. The treatment protocol of group II patients did not include ACE inhibitors, while some patients in both groups continued to receive class II antiarrhythmic drugs, diuretics, or their combinations. This treatment was continued until the day before the operation. Patients in both groups were followed up in the postoperative period (until discharge) with clinical and hematologic parameters in terms of AKI and some factors (such as mortality rates, blood values showing renal function, need for dialysis, amount of bleeding and revision, intensive care and hospital stay, organ dysfunctions, and hospital costs). In addition, even if the patients did not have any complaints in the post-discharge period, they were called to the hospital for follow-up and evaluated for AKI within the first 2 years (for patients who could be reached).

Cardiac surgery procedures were performed using conventional CPB and cross-clamping for the surgical procedure. After the placement of peripheral arterial and venous catheters, general anesthesia and endotracheal intubation were performed, and all patients underwent a median sternotomy. The internal mammarian artery and saphenous veins were used for cardiac revascularization. Mechanical and biological valves were available for patients with valvular heart disease. Synthetic tube grafts were also provided for patients undergoing aortic surgery. After heparinization (active clotting time over 400 seconds), aortic and right atrial cannulas were placed to connect patients to CPB. Patients were connected to the heart-lung machine, and CPB was started. Body temperature was reduced to 28°C to 32°C, and cardioplegic solution was infused into the aortic root following aortic clamping in all patients. The pulse pump flow rate was maintained at 1.5 to 2.4 liters per minute. Patients were weaned from CPB after surgical procedures, and systemic warming and hemodynamic stability were achieved.

AKI is a common syndrome that is independently associated with increased mortality. A standardized definition is important to facilitate clinical care and research. The definition of AKI has evolved rapidly since 2004, with the introduction of the risk, injury, failure, loss, and end-stage renal disease (RIFLE), AKI network (AKIN), and kidney disease improving global outcomes (KDIGO) classifications. The recent definition from the international KDIGO guideline merged RIFLE and AKIN. A systematic review has found that these definitions do not differ significantly in their performance. These efforts to standardize the AKI definition are a substantial advance, although areas of uncertainty remain. AKI is usually diagnosed when the serum creatinine (Cr) level has increased by >1.5 times the baseline value within the last 7 days, a decrease in urine output over a fixed period, or when the GFR has decreased by >25%.^[9,10]^

In this article, we considered the definition criteria for AKI in the literature as indicators of renal damage. In addition, we also evaluated the need for dialysis, creatinine clearance (CrCL) change, and blood nitrogen urea (BUN) levels in our patients. Thus, in the light of prospectively collected data recorded by cardiac surgeons, AKI was diagnosed and renal damage was evaluated in the light of the parameters listed below.

1. Postoperative renal failure requiring dialysis before discharge
2. Decreases of 25% or higher in the GFR and CrCL <80 mL/min/1,73 m^2^ without requiring dialysis
3. BUN levels are >50 mg/dL, and creatinine levels have increased by >1.5 times the baseline value within the last 7 days.

Routine clinical monitoring included a central venous catheter and a femoral or radial artery catheter. Systemic arterial pressure and central venous pressure were constantly measured with disposable transducers. Cardiac output was continuously measured: A high level of accuracy and precision has previously been demonstrated between the continuous cardiac output measurements and the intermittent manual bolus thermodilution technique.^[11,12]^ The serum creatinine, creatinine clearance, blood urea nitrogen, and GFR levels were measured at baseline and at the 12^th^, 24^th^, 48^th^, 72^nd^, and 5^th^ days after cardiac surgery. The clearances during these time intervals were calculated according to the standard formulae. Patients who did not benefit sufficiently from renal replacement therapy, whose renal destruction was proven according to AKI definition criteria, whose uremic symptoms became evident, and whose renal damage was supported by biochemical blood tests (such as acidosis and hyperkalemia), were included in the hemodialysis program without delay based on KDIGO criteria.^[13,14]^

Preoperative data included age, gender, history of diabetes mellitus, arterial vascular disease, hypertension (history of hypertension requiring medical treatment), history of MI, and smoking. Hemodynamic variables were measured or calculated before the induction of anesthesia. Surgical procedures performed were classified as coronary artery bypass graft isolated or combined with valve surgery, isolated valve repair or replacement surgery, adult congenital procedures, and aortic surgery. Data on operative events included operative time, total CPB, and cross-clamp times. After completion of surgery, inotrope vasopressor and nitro-dilator infusions and intra-aortic balloon pump (IABP) insertions in the ICU were recorded. Biochemical, hematologic, and microbiologic laboratory data and the length of hospital stay in the ICU were obtained from medical records. In addition to digital glycosides, catecholamine, loop diuretics, antiarrhythmics (xylocaine, amiodarone), and intravenous glyceryl trinitrate at standard doses were allowed throughout the study. Permission was obtained from the Hospital Ethics Committee before starting the study. In addition, all procedures were performed in accordance with the Declaration of Helsinki.

Informed consent was obtained and signed by the patient and the patient’s first degree relatives. Exclusion criteria included emergency and repeat surgeries, renal artery stenosis, hypertrophic cardiomyopathy, prior coronary artery bypass graft, use of nonsteroidal anti-inflammatory drugs, severe anemia, active infection, severe hepatic impairment, or prior AKI (creatinine level has increased by >1.5 times the baseline value, CrCL levels <80 mL/min/1.73m^2^).

Operative mortality includes the first day of the postoperative period. Overall mortality was defined as death from any cause during hospitalization or until hospital discharge. The length of ICU stays and hospital discharge days in both groups were compared. Hospital charges were obtained through the hospital billing department, as reported to our hospital authorities. These represent all hospital charges for the index hospitalization only and include charges incurred during surgery. The cost data is broken down into the following categories: routine costs, operating room facility usage, operating room supply usage, medication costs, laboratory costs, radiology costs, physical therapy costs, etc. Disposable supply costs are based on actual purchase costs. Labor costs for nurses, technicians, fellows, assistants, secretaries, orderlies, and other staff are derived directly from actual salaries and include benefits. All costs are calculated per patient and presented in US dollars. In general, the cost of a cardiac surgery patient in our hospital was around 3000 dollars. Based on this price, the costs between the 2 groups were compared. Patients were followed up for AKI for 2 years after discharge (even if serum creatinine levels were in the normal range before discharge), and readmission rates were compared between the 2 groups.

Neurological complications were defined as any transient or permanent neurological deficit that developed after surgery. Gastrointestinal complications included a confirmed diagnosis of upper and lower gastrointestinal hemorrhage, intestinal ischemia, acute cholecystis, and pancreatitis. Generally, mortality, per-operative acute myocardial infarction, IABP usage, incidence of LCOS, renal failure, use of inotropic agents, ICU and hospital stay, cardiac hemodynamic changes, bleeding, revision rates, gastrointestinal, pulmonary, and neurological complications, infections, and survival rates were determined.

### 2.1. Statistical analysis

Qualitative data are presented as numbers and percentages, and measurement data are presented as the arithmetic mean ± standard deviation for all continuous variables. The Mann-Whitney test was used for continuous variables, and Wilcoxon signed rank test was used to compare preoperative and postoperative variables within the same group. Categorical variables were expressed as percentages as well as actual numbers and compared using the x^2^ or Fisher exact test. Statistically significant differences were noted for each analysis, and statistical significance was based on a *P*-value <.05. Statistical analyses were performed using SPSS 11 software (SPSS, Chicago).

## 3. Results

293 patients (47.9%) did not receive ACE inhibitor therapy prior to surgery, while 319 patients (52.1%) did. The groups exhibited comparable values for age, weight, gender distribution, smoking status, arterial vascular disease, preoperative GFR, and history of myocardial infarction. Table 1 shows the preoperative demographic characteristics. ACE inhibitors were to be used by patients at an appropriate dose starting at least 4 weeks before surgery (one oral dose per day). There was no significant difference between the groups in terms of preoperative characteristics and β-blocker use (Table 1). CrCL, creatinine, blood urea nitrogen levels, and preoperative hematocrit exhibited no significant differences between the 2 cohorts. Regarding systemic hemodynamic variables and preoperative medications, no statistically significant differences were observed (*P* >.05).

**Table 1:** Preoperative patient characteristics among groups.

Variables	Group I (n = 319)	Group II (n = 293)	*P*-value
Median age	61 ± 4.1	62 ± 4.8	.198
Sex			
Male	162 (50.8%)	150 (51.2%)	.499
Female	157 (49.2%)	143 (48.8%)	
Median weight (kg)	77 ± 5.7	75 ± 6.1	.255
Diabetes mellitus	95 (29.8%)	88 (30%)	.135
COPD	101 (31.6%)	105 (35.8%)	.289
Smoking	258 (80.8%)	241 (82.2%)	.901
Hematocrit	47 ± 4.4	48 ± 5.6	.236
Hypertension	138 (43.2%)	127 (43.3%)	.701
Preoperative IABP	34 (10.6%)	32 (109%)	.733
Preoperative median GFR	167 ± 25	162 ± 27	.339
PAD	57 (17.8%)	52 (17.7%)	.400
History of MI	101 (31.6%)	107 (36.5%)	.108
LVEF <0.40	57 (17.8%)	55 (18.7%)	.705
BUN levels (mg/dL)	18 ± 6.1	19 ± 7	.520
Creatinine levels (mg/dL)	1.0 ± 0.31	0.9 ± 0.42	.117
CrCL (mL/min)	51 ± 10.8	49 ± 11.1	.750
Use of β-blockers	244 (76.5%)	237 (80.8%)	.498
Use of diuretics	102 (31.9%)	110 (37.5%)	.321
Use of antiarrhythmic	30 (9.4%)	25 (8.5%)	.433
Mean pulse rate (beats/min)	69 ± 16.8	72 ± 15.4	.288
Mean systolic BP (mm Hg)	101 ± 12.8	98 ± 13.1	.622
Mean CVP (mm Hg)	8 ± 1.4	7 ± 1.5	.899
Mean PAP (mm Hg)	18 ± 10.8	19 ± 11.1	.522
Mean CI (L/min/m^2^)	2.2 ± 0.4	2.3 ± 0.5	.316

BP = blood pressure, CI = cardiac Index, COPD = chronic obstructive pulmonary disease, CrCL = creatinine clearance, CVP = central venous pressure, GFR = glomerular filtration rate, IABP = intra-aortic balloon pump, LVEF = left ventricular ejection fraction, MI = myocardial infarction, PAD = peripheral arterial disease, PAP = pulmonary artery pressure.

Table 2 demonstrates that surgical procedures, cross-clamping time, number of distal anastomoses, cardiac drug use, and extubation periods were commensurate between the 2 groups (*P* >.05). Postoperative IABP was required in 7 patients (in addition to preoperative patients) in both groups. No patient required extracorporeal membrane oxygenation (ECMO) due to the low flow rate. There was no difference between the groups in terms of the number of vessels bypassed, arterial conduit type, or surgical anastomosis sites. There was also no difference in the use of the internal mammarian artery between the groups. There was also no statistical difference in the duration of surgery or CPB between the groups. The cardiovascular surgery-related characteristics of the groups are shown in Table 2.

**Table 2:** Operative data.

Data	Group I (n = 319)	Group II (n = 293)	*P*-value
Only CABG	194 (60.8%)	185 (63.1%)	.563
Only MVR	57 (17.8%)	52 (17.7%)	.788
Only AVR	30 (9.4%)	27 (9.2%)	.322
CABG + MVR	12 (3.7%)	10 (3.8%)	.404
CABG + AVR	13 (4%)	10 (3.4%)	.336
Aortic surgery	8 (2.5%)	6 (2%)	.569
ASD	5 (1.6%)	3 (1%)	.336
CC time (min)	44 ± 11.8	45 ± 11.4	.566
CPB time (min)	102 ± 10.2	110 ± 11.1	.216
Operating time (h)	3.5 ± 1.2	3.7 ± 1.3	.265
Distal anastomoses	3.2 ± 1.5	3.1 ± 1.3	.416
Postoperative IABP	41 (12.8%)	39 (13.3%)	.155
IMA usage	184 (99%)	179 (99.4%)	.933

ASD = atrial septal defect, AVR = aortic valve replacement, CABG = coronary artery bypass grafting, CC = cross clamping, CPB = cardiopulmonary bypass, IABP = intra-aortic balloon pump, IMA = internal mammarian artery, MVR = mitral valve replacement.

Group II exhibited considerably elevated levels of blood urea nitrogen and creatinine postoperatively in comparison to their preoperative values (*P* <.05). The reductions in creatinine clearance and GFR were significantly greater in the control group than in the treatment group (*P* <.05). 31 patients in the control group required dialysis, whereas only 9 patients in the treatment group did so (*P* <.05). During the follow-up within 2 years, no other patients in the drug treatment group required dialysis in addition to the patients who required early dialysis. Among the patients in Group II, in addition to the 31 patients who required dialysis in the postoperative period, 15 patients also required dialysis (Table 3). In conclusion, renal damage, AKI, and the need for dialysis after cardiac surgery were higher in the control group compared to the treatment group.

**Table 3:** Postoperative data. *P*-values of <.05 were considered significant.

Data	Group I (n = 319)	Group II (n = 293)	*P*-value
Use of inotropes	266 (83.3%)	274 (93.5%)	.256
Use of trinitrat	28 (8.7%)	33 (11.2%)	.777
Use of nipruss	12 (3.7%)	20 (6.8%)	.563
Extubation time (h)	3.1 ± 1.5	3.2 ± 1.2	.412
Postoperative BUN levels (mg/dL)	**15 ± 9.5**	**45 ± 10.8**	**<.05**
Postoperative creatinin levels (mg/dL)	**1.0 ± 0.3**	**2.2 ± 1.5**	**<.05**
Postoperative CrCL (mL/min)	**39 ± 14.1**	**15 ± 13.4**	**<.05**
Postoperative median GFR	**145 ± 20.5**	**82 ± 21.5**	**<.05**
Requiring dialysis	**9 (2.8%**)	**31 (10.6%**)	**<.05**
ICU stay	**2.1 ± 1.8**	**6.2 ± 1.7**	**<.05**
Hospital stay	**5.1 ± 2.2**	**14.7 ± 3.1**	**<.05**
Surgical revision for blood loss	12 (3.7%)	10 (3.7%)	.567
Postoperative blood loss >1000 mL	51 (15.9%)	55 (18.7%)	.971
Infection	65 (3.7%)	61 (5.1%)	.215
Operative mortality (first 24 h)	6 (3.2%)	8 (4.4%)	.127

BUN = blood urea nitrogen, CrCL = creatinine clearance, GFR = glomerular filtration rate, ICU = intensive care unit.

There was no difference between the groups in terms of systemic dysfunction, infection, blood volume, or death. The study found that the operating mortality rate was 3.2% among patients who underwent preoperative ACE inhibitor medication, while it was 4.4% among those who did not receive this therapy (*P* >.05) (Table 4). Low cardiac output was responsible for most deaths in the first 24 hours. There was no difference between the groups of these patients. All of these patients were on IABP before and after surgery. There was no statistically significant difference in overall mortality between the groups. During follow-up within 2 years, no significant difference was detected in terms of patient loss.

**Table 4:** The clinical results after cardiac surgery (in period early and late). *P*-values of <.05 were considered significant.

Postoperative results	Group I (n = 319)	Group II (n = 293)	*P*-value
Cardiac dysfunction	22 (6.9%)	20 (6.8%)	.231
Cardiac arrhythmia	40 (12.5%)	44 (15%)	.745
Pulmonary dysfunction	39 (12.2%)	41 (14%)	.556
Gastrointestinal system dysfunction	4 (1.2%)	3 (1%)	.333
Hepatic dysfunction	3 (0.9%)	3 (1%)	.598
Neurological dysfunction	11 (3.4%)	14 (4.7%)	.367
Multiple organ dysfunction	2 (0.6%)	3 (1%)	.789
Nosocomial infection	34 (10.6%)	31 (10.5%)	.578
Urinary tract infection	11 (3.4%)	10 (3.4%)	.127
Mediastinal infection	5 (1.5%)	4 (1.3%)	.697
Pneumonia	15 (4.7%)	16 (5.4%)	.941
Time to extubation (h)	2.7 ± 1.1	2.6 ± 1.2	.378
Surgical revision for bleeding	11 (3.4%)	10 (3.4%)	.480
Postoperative bleeding >1000 mL/24 h	28 (8.7%)	32 (10.9%)	.411
Overall death (until discharge time)	7 (2.2%)	8 (2.7%)	.571
Hospital costs (over $3000)	**16 (5.0%**)	**77 (26.3%**)	**<.05**
Within 2 yr			
Patient death	8 (2.5%)	12 (4.0%)	.125
Hospital readmissions	**5 (1.5%**)	**88 (30%**)	**<.05**
Requiring dialysis	**0**	**15 (5.1%**)	**<.05**

A notable disparity was seen among the groups with regards to the duration of stay in the ICU and the hospital. The duration of ICU and hospitalization was found to be shorter in Group I compared to Group II, and this finding was determined to be statistically significant (*P* <.05) (Table 3). In most patients who were not given ACE inhibitors, hospital costs exceeded $3000 by the day of discharge. There was a statistically significant difference between the 2 groups in this respect (*P* <.05). In both groups of patients who were followed up for AKI for 2 years after discharge, the rate of re-hospitalization for kidney injury was significantly higher in group II patients. This difference was statistically significant (*P* <.05).

## 4. Discussion

The administration of an ACE inhibitor to patients having cardiac surgery with CPB support who do not have preexisting cardiac or renal failure has the potential to enhance renal function.^[3,4,14]^ Generally, ACE inhibitors have been observed to enhance renal blood flow and reduce renal vascular resistance.^[14,15]^ Consequently, the administration of ACE inhibitors during cardiac surgery has been associated with favorable outcomes in renal function. Acute renal failure following cardiac surgery with CPB support is a significant challenge that is difficult to overcome. There is still some risk of postoperative adverse events associated with postoperative AKI, even with the use of improved anesthetic, surgical, and cardiac protection procedures. The potential advantages have been derived from several characteristics associated with the administration of ACE inhibitors.^[1,16]^ It has been asserted that ACE inhibitors have the ability to reinstate the integrity of the vascular endothelium, a crucial factor in ensuring sufficient blood flow in the microvasculature and the maintenance of organ function.

The administration of ACE inhibitors has been associated with a potentially reversible decline in renal function in various clinical scenarios, including heart failure, the concurrent use of nonsteroidal anti-inflammatory drugs, renal artery stenosis, inadequate fluid volume, hypertrophic cardiomyopathy, and cardiopulmonary bypass, leading to significant impairment of renal blood flow.^[5,16]^ These conditions were deemed ineligible for inclusion in our study. This study demonstrates a statistically significant association between the administration of preoperative ACE inhibitors and a reduced incidence of postoperative renal failure in comparison with those who did not receive preoperative ACE inhibition medication. This study of ours found a significant reduction in creatinine and blood urea nitrogen levels in the treatment group as a result of ACE inhibitor administration. In addition, it was observed that the control group consistently exhibited higher postoperative creatinine clearance and GFR compared to the treatment group. Additionally, there was a greater demand for dialysis in the control group. In addition, in the evaluation of the patients who were followed up for 2 years and brought for follow-up, the rate of deterioration in renal function was much less in patients who received ACE inhibitors preoperatively compared to the group who did not receive ACE inhibitors. And the need for new dialysis was also absent in group I patients, whereas it was statistically high in group II patients. In contrast to our research findings, certain publications have reported a lack of observed enhancement in renal function linked to preoperative ACE inhibitor use.^[1,17]^ The observed discrepancy in outcomes between the 2 trials may be attributed to variations in the characteristics of the surgical patient populations and discrepancies in the criteria used to define renal impairment within each respective study. Furthermore, the findings of our study have received validation from several researchers. It was shown that administering a dose of an intravenous ACE inhibitor resulted in an enhancement of renal perfusion through the inhibition of renin-angiotensin system activity. Additionally, ACE inhibitors were observed to elevate cardiac output in individuals with left ventricular failure, thereby leading to an improvement in renal perfusion.^[3,18]^ The observed reduction in the incidence of AKI among patients who were administered ACE inhibitors prior to surgery may be attributed to the renal perfusion-protective effect of ACE inhibition during the intraoperative period. In addition, the administration of ACE inhibitors prior to surgery in the surgical population may potentially be linked to an increased preoperative renal function reserve. This could potentially reduce the vulnerability of these patients to renal injury during subsequent cardiopulmonary bypass. According to previous research,^[17–19]^ the administration of ACE inhibitors as a preoperative long-term medication may result in the occurrence of hypotension both before and after surgery. No negative effects were observed in individuals receiving ACE inhibitors, such as hypotension during anesthesia induction or an elevated requirement for vasoconstrictors in the short and long term following CPB.

Certain clinical outcome markers, including the frequency of single or multiple organ failure, prolonged mechanical breathing, length of ICU stay, and death following cardiovascular surgery, have not been found to be affected by ACE inhibitors, according to certain studies.^[1]^ Treatment with ACE inhibitors may help with the remodeling of cardiac myocytes and vascular smooth muscle, as well as the regression of hypertrophic alterations. The decreased adrenergic sensitivity of the cardiovascular system following ACE inhibitor treatment has been explained by structural alterations in end organs.^[20,21]^ However, there was no difference observed in this trial between preoperative ACE inhibitor medication and postoperative hemodynamic measures or cardiovascular support with inotropes and vasopressors. ACE inhibitor therapy initiated at least 1 month before surgery was well tolerated by patients and significantly reduced the rates of AKI and the need for dialysis in the postoperative period. Furthermore, this study showed that these agents significantly reduced the length of hospitalization, readmission rates, and total cost of hospitalization.

With ACE inhibitors used before cardiac surgery, the intensive care, hospitalization, and readmission times of patients were within normal limits. In the untreated patient group, these durations and readmission rates were found to be high, and hospital costs increased accordingly. The operation cost for uncomplicated cardiac surgery in our hospital is approximately 3000 dollars. If an additional problem, such as AKI or the need for dialysis, occurs in patients, the length of hospital stays increases and the cost exceeds $3000. In our study, hospital costs until the day of discharge for patients who underwent cardiac surgery without drug therapy were over $3000. In addition, the incidence of postoperative AKI and dialysis was lower, ICU and hospital stays were shorter, and readmission rates and hospital costs were lower in the drug group (Table 4). Our results were consistent with the results of the KDIGO Diabetes Work Group study and the study of Khwaja A et al and are consistent with the relevant literature.^[13,14]^

The present study is subject to many limitations. Initially, it is important to note that this study did not employ a randomized design. Hence, it is plausible that our findings could have been influenced by treatment bias. The clinical findings of this investigation were limited in validity due to their retrospective nature and the small sample size, despite the utilization of certain statistical analyses that facilitated a relatively accurate evaluation and comparison of risks and outcomes. Further research is warranted to monitor these patients, particularly in order to ascertain the potential advantages of using ACE inhibitors as a preoperative therapy. While it is essential to have a larger sample size to ensure more statistically significant findings, our investigation may already yield intriguing data pertaining to preoperative ACE inhibitors. Moreover, our analysis encompasses a wide range of surgical procedures related to cardiac surgery operations on CPB. Consequently, it is important to acknowledge that our findings may be influenced by many systemic alterations and pathological states that occur during and following cardiac surgery.

In conclusion, the use of ACE inhibitors prior to cardiac surgery was associated with a significant improvement in renal function. Optimal management of ACE inhibitor therapy prior to cardiac surgery may help prevent AKI and favorably impact the clinical sequelae of cardiopulmonary bypass and cardiac surgical procedures. In addition, in patients who do not receive ACE inhibitor therapy, postoperative kidney injury and high morbidity rates may contribute to prolonged hospitalization, readmission, and increased surgical costs.

## Acknowledgments

We would like to thank the clinical assistants for obtaining, recording and collating the patients’ data.

## Author contributions

**Conceptualization:** Yasin Kilic, Ebubekir Sonmez, Bilgehan Erkut.

**Data curation:** Yasin Kilic, Izatullah Jalalzai, Ebubekir Sonmez, Bilgehan Erkut.

**Formal analysis:** Yasin Kilic, Bilgehan Erkut.

**Funding acquisition:** Yasin Kilic, Izatullah Jalalzai.

**Investigation:** Yasin Kilic, Bilgehan Erkut.

**Methodology:** Yasin Kilic.

**Project administration:** Yasin Kilic, Izatullah Jalalzai, Bilgehan Erkut.

**Resources:** Yasin Kilic, Izatullah Jalalzai, Ebubekir Sonmez, Bilgehan Erkut.

**Software:** Yasin Kilic, Ebubekir Sonmez.

**Supervision:** Yasin Kilic, Izatullah Jalalzai, Bilgehan Erkut.

**Validation:** Yasin Kilic, Izatullah Jalalzai.

**Visualization:** Yasin Kilic.

**Writing – original draft:** Yasin Kilic, Izatullah Jalalzai, Ebubekir Sonmez, Bilgehan Erkut.

**Writing – review & editing:** Yasin Kilic, Izatullah Jalalzai, Ebubekir Sonmez, Bilgehan Erkut.

## References

[1] Rady MY Ryan T. The effects of preoperative therapy with angiotensin-converting enzyme inhibitors on clinical outcome after cardiovascular surgery. Chest. 1998;114:487–94. 9726735 10.1378/chest.114.2.487

[2] Baue AE. Multiple organ failure, multiple organ dysfunction syndrome and systemic inflammatory response syndrome: why no magic bullets? Arch Surg. 1997;132:703–7. 9230852 10.1001/archsurg.1997.01430310017002

[3] Ryckwaert F Colson P Ribstein J Boccara G Guillon G. Haemodynamic and renal effects of intravenous enalaprilat during coronary artery bypass graft surgery in patients with ischaemic heart dysfunction. Br J Anaesth. 2001;86:169–75. 11573655 10.1093/bja/86.2.169

[4] Benedetto U Sciarretta S Roscitano A. Preoperative angiotensin-converting enzyme inhibitors and acute kidney injury after coronary artery bypass grafting. Ann Thorac Surg. 2008;86:1160–5. 18805152 10.1016/j.athoracsur.2008.06.018

[5] Mann JF Gerstein HC Yi QL. Development of renal disease in people at high cardiovascular risk: results of the HOPE randomized study. J Am Soc Nephrol. 2003;14:641–7. 12595499 10.1097/01.asn.0000051594.21922.99

[6] Marre M Lievre M Chatellier G Mann JF Passa P Ménard J; DIABHYCAR Study Investigators. Effects of low dose ramipril on cardiovascular and renal outcomes in patients with type 2 diabetes and raised excretion of urinary albumin: randomised, double blind, placebo controlled trial (the DIABHYCAR study. BMJ. 2004;328:495–8. 14960504 10.1136/bmj.37970.629537.0D PMC351842

[7] Gameiro J Fonseca JA Outerelo C Lopes JA. Acute kidney injury: from diagnosis to prevention and treatment strategies. J Clin Med. 2020;9:1704. 32498340 10.3390/jcm9061704 PMC7357116

[8] Hoste EA Bagshaw SM Bellomo R. Epidemiology of acute kidney injury in critically ill patients: the multinational AKI-EPI study. Intensive Care Med. 2015;41:1411–23. 26162677 10.1007/s00134-015-3934-7

[9] Thomas ME Blaine C Dawnay A. The definition of acute kidney injury and its use in practice. Kidney Int. 2015;87:62–73. 25317932 10.1038/ki.2014.328

[10] Rossaint J Zarbock A. Acute kidney injury: definition, diagnosis and epidemiology. Minerva Urol Nefrol. 2016;68:49–57. 26364570

[11] Bakris GL Weir MR. Angiotensin-converting enzyme inhibitor- associated elevations in serum creatinine: is this a cause for concern? Arch Intern Med. 2000;160:685–93. 10724055 10.1001/archinte.160.5.685

[12] Ostermann M Kunst G Baker E Weerapolchai K Lumlertgul N. Cardiac surgery associated AKI prevention strategies and medical treatment for CSA-AKI. J Clin Med. 2021;10:5285. 34830567 10.3390/jcm10225285 PMC8618011

[13] Kidney Disease: Improving Global Outcomes (KDIGO) Diabetes Work Group. KDIGO 2020 clinical practice guideline for diabetes management in chronic kidney disease. Kidney Int. 2020;98(4S):S1–S115. 32998798 10.1016/j.kint.2020.06.019

[14] Khwaja A. KDIGO clinical practice guidelines for acute kidney injury. Nephron Clin Pract. 2012;120:c179–84. 22890468 10.1159/000339789

[15] Ostermann M Cennamo A Meersch M Kunst G. A narrative review of the impact of surgery and anaesthesia on acute kidney injury. Anaesthesia. 2020;75:e121–33. 31903567 10.1111/anae.14932

[16] Zarbock A Küllmar M Ostermann M. Prevention of cardiac surgery-associated acute kidney injury by implementing the KDIGO guidelines in high-risk patients identified by biomarkers: the PrevAKI-multicenter randomized controlled trial. Anesth Analg. 2021;133:292–302. 33684086 10.1213/ANE.0000000000005458

[17] Gamoso MG Phillips-Bute B Landolfo KP Newman MF Stafford-Smith M. Off-pump versus on-pump coronary artery bypass surgery and postoperative renal dysfunction. Anesth Analg. 2000;91:1080–4. 11049887 10.1097/00000539-200011000-00007

[18] Wagner F Yeter R Bisson S Siniawski H Hetzer R. Beneficial hemodynamic and renal effects of intravenous enalaprilat following coronary artery bypass surgery complicated by left ventricular dysfunction. Crit Care Med. 2003;31:1421–8. 12771613 10.1097/01.CCM.0000063050.66813.39

[19] Chou YH Huang TM Wu VC; National Taiwan University Study Group on Acute Renal Failure (NSARF). Associations between preoperative continuation of renin-angiotensin system inhibitor and cardiac surgery-associated acute kidney injury: a propensity score-matching analysis. J Nephrol. 2019;32:957–66. 31595420 10.1007/s40620-019-00657-4

[20] Pickkers P Darmon M Hoste E. Acute kidney injury in the critically ill: an updated review on pathophysiology and management. Intensive Care Med. 2021;47:835–50. 34213593 10.1007/s00134-021-06454-7 PMC8249842

[21] Sharif S Chen B Brewster P Chen T Dworkin L Gong R. Rationale and design of assessing the effectiveness of short-term low-dose lithium therapy in averting cardiac surgery-associated acute kidney injury: a randomized, double blinded, placebo controlled pilot trial. Front Med. 2021;8:639402. 10.3389/fmed.2021.639402 PMC8236527 34195206

